# Evidence for Public Health Risks of Wastewater and Excreta Management Practices in Southeast Asia: A Scoping Review

**DOI:** 10.3390/ijerph121012863

**Published:** 2015-10-15

**Authors:** Steven Lam, Hung Nguyen-Viet, Tran Thi Tuyet-Hanh, Huong Nguyen-Mai, Sherilee Harper

**Affiliations:** 1 Department of Population Medicine, University of Guelph, Guelph 50 Stone Rd. E., Guelph, ON N1G 2W1, Canada; 2 Center for Public Health and Ecosystem Research, Hanoi School of Public Health, 138 Giang Vo. Street, Hanoi 10000, Vietnam; E-Mails: h.nguyen@cgiar.org (H.N.-V.); maihuong.hsph7@gmail.com (H.N.-M.); 3 International Livestock Research Institute (ILRI), 17A Nguyen Khang Street, Trung Hoa Ward, Cau Giay District, Hanoi 10000, Vietnam; 4 Swiss Tropical and Public Health Institute, 57 Socinstrasse, CH-4002 Basel, Switzerland and University of Basel, Basel CH-4002, Switzerland; 5 Department of Environmental Health, Hanoi School of Public Health, 138 Giang Vo. Street, Hanoi 10000, Vietnam; E-Mail: tth2@hsph.edu.vn

**Keywords:** health risks, scoping review, Southeast Asia, wastewater management, excreta management, agricultural intensification

## Abstract

The use of wastewater and excreta in agriculture is a common practice in Southeast Asia; however, concerns remain about the potential public health risks of this practice. We undertook a scoping review to examine the extent, range, and nature of literature, as well as synthesize the evidence for associations between wastewater and excreta management practices and public health risks in Southeast Asia. Three electronic databases (PubMed, CAB Direct, and Web of Science) were searched and a total of 27 relevant studies were included and evaluated. The available evidence suggested that possible occupational health risks of wastewater and excreta management practices include diarrhea, skin infection, parasitic infection, bacterial infection, and epilepsy. Community members can be at risk for adverse health outcomes through consuming contaminated fish, vegetables, or fruits. Results suggested that practices including handling, treatment, and use of waste may be harmful to human health, particularly farmer’s health. Many studies in this review, however, had limitations including lack of gender analyses, exposure assessment, and longitudinal study designs. These findings suggest that more studies on identifying, quantitatively assessing, and mitigating health risks are needed if sustainable benefits are to be obtained from wastewater and excreta reuse in agriculture in Southeast Asia.

## 1. Introduction

Population and economic growth has led to rapid agricultural intensification in Southeast Asia (Brunei, Cambodia, Indonesia, Laos, Malaysia, Myanmar, Philippines, Singapore, Thailand, Timor Leste, and Vietnam). While such agricultural change can be beneficial for human health through increased food security and socio-economic development, agricultural intensification also can be detrimental to human health through increased pathogen virulence and facilitation of new zoonoses [1,2]. The public health impacts of agricultural intensification in Southeast Asia are not yet well understood [1]. For instance, untreated wastewater as well as animal and human excreta are commonly used in agriculture and aquaculture in Southeast Asia [3,4], as they can provide good sources of nutrients for crops and thus reduce the need for commercial fertilizers [5,6,7]; however, agricultural intensification is resulting in increased livestock numbers leading to generation of excreta and wastewater in large quantities in constrained areas. While wastewater and excreta are valuable resources for farmers, the increased livestock production combined with outdated technologies and management practices may present substantial human health and environmental health concerns [8,9,10]. As such, the potential impact of intensive agriculture and related livestock production on human health is a growing concern [1,11].

Untreated wastewater and excreta often contain many different types of contaminants that can impact human health, including excess nutrients, pathogens, heavy metals, and hormones [8,12]. Possible groups who are at risk of exposure to these contaminants include farm workers, their families, consumers, and nearby community members [13]. Occupational exposure to contaminants can occur through direct contact with untreated wastewater and excreta via waste management practices including the handling, storage, treatment, use, and disposal of wastewater or excreta. Those living nearby intensive agricultural operations can be exposed to contaminants indirectly, for example, contaminants can enter drinking water sources through the application of untreated wastewater and excreta to agricultural fields [14]. Overapplication of untreated wastewater and excreta can also lead to runoff and overland flow after rainfall events, which can result in the contamination of surface water, as well as untreated wastewater and excreta leaching through permeable soils and contaminating vulnerable aquifers [8]. Wastewater and excreta management practices may cause environmental problems including pollution of surface water, groundwater, and soil by nutrients, pathogens, organic matter, and heavy metals, as well as emissions of ammonia, odour, and greenhouse gases [9,15,16]. Considering the wide range of contaminants and different exposure routes, concern remains about the potential human health risks [8,13,17,18]. Epidemiologically, it is important to differentiate between the potential risk of exposure, confirmed exposure to contaminants, and confirmation that exposure to contaminants resulted in illness. For instance, an individual may be at risk of exposure to contaminants because they live near an intensive agricultural operation, but never come in contact with the contaminant; similarly, an individual who is exposed to contaminants might not develop an illness. Whether or not an individual develops an illness depends on a variety of host (e.g., lifestyle, behavior, vulnerable populations), environment (e.g., environmental sanitations, provisions of safe water or disposal of excreta), and agent factors (e.g., infectious dose, infection causes disease or furthers transmission). It is unclear, however, about the most important protective and risk factors associated with different illnesses in humans.

Encouraging the safe use of wastewater and excreta in agriculture and aquaculture has important economic and environmental benefits, such as conserving water and recycling nutrients. Although the potential human and environmental health risks related to wastewater and excreta use are generally known, the epidemiological evidence regarding the associations between poor health outcomes and wastewater and excreta management practices in Southeast Asia have not been synthesized. As such, there is a need to examine and clarify the strength of evidence about the potential human health risks of wastewater and excreta management, as to provide a useful resource to policy-makers and to inform future research in the region. Thus, the objective of this scoping review was to identify and synthesize the extent, range, and nature of evidence of public health risks posed by wastewater and excreta management practices in Southeast Asia.

## 2. Methods

A scoping review approach was used, which aims to map the existing literature supporting broad research questions on a specific topic [19]. The methodology for this scoping review was based on the framework outlined by Arksey and O’Malley [19]. The review included the following five key phases: (1) identifying the research question; (2) identifying relevant studies; (3) study selection; (4) charting the data; and (5) collating, summarizing, and reporting the results.

### 2.1. Research Question

This review was guided by the question “What are the human health risks of wastewater, human excreta, and animal excreta management practices in Southeast Asia? The acronym PICO was used to frame the research question according to Population (e.g., people in Southeast Asia), Intervention (e.g., wastewater or excreta management practice), Comparison (e.g., no wastewater or excreta management practice), and Outcome (e.g., disease, illness, or poor health).

### 2.2. Data Sources and Search Strategy

The initial search was implemented on February 21, 2015 in three electronic databases: PubMed (http://www.ncbi.nlm.nih.gov/pubmed/), CAB Direct (http://cabdirect.org), and Web of Science (http://webofscience.com). These databases were selected to be comprehensive and to cover disciplines in health, agriculture, and environment. Limits on database search included peer-reviewed literature in the English language published from 1 January 2000 to 31 December 2014. The date range limitation was chosen in order to focus on the most recent literature on waste management in the 21st century. The search strategy employed broad search terms (Table 1) to ensure publications were not overlooked, and many publications were then excluded. The reference lists of all relevant articles were hand-searched to identify any further relevant studies not captured in the database search.

**Table 1:** Scoping review search strategy with algorithms for each database to identify peer-reviewed articles examining the human health risks of wastewater, human excreta, and animal excreta management practices in Southeast Asia.

Databases	Main Terms	Expanded Terms
PubMed, CAB Direct, Web of Science	Health effects	“adverse effect” OR health OR disease OR death OR morbidity OR mortality OR pathogen OR illness OR ailment OR allerg* OR zoonos* OR infection OR diarrhea OR “well-being” OR “well being” AND
	Waste management	“agricultural waste” OR wastewater OR “waste water” OR “integrated waste” OR “faecal sludge” OR manure OR “animal waste” OR “solid waste” OR “human waste” OR “livestock waste” OR faeces OR feces OR “animal waste” OR excreta OR excrement AND
	Location	Brunei OR Cambodia OR Indonesia OR Laos OR Malaysia OR Myanmar OR Philippines OR Singapore OR Thailand OR “Timor Leste” OR “Viet Nam” OR Vietnam OR “Southeast Asia” OR “South East Asia”

### 2.3. Citation Management

All citations were imported into the web-based application DistillerSR (Evidence Partners Incorporated, Ottawa, ON, Canada) and duplicate citations were removed using the DistillerSR duplicate removal function. Title and abstract relevance screening and data characterization of full articles were subsequently performed using DistillerSR.

### 2.4. Relevance Screening and Eligibility Criteria

A two-step relevance screening strategy was employed. For the first level of screening, titles and abstracts of articles were screened for relevance. Next, all citations deemed relevant after title and abstract screening went through review of the full-text articles. Studies were eligible for inclusion if they were original articles on wastewater or excreta management relevant to human health, environmental health, or perceived health risks (Table 2). The title and abstract, as well as full-text of each citation were independently screened by two reviewers (Steven Lam, Anna Manore). Then, the reference list of all included articles was hand-searched for other relevant articles. Reviewers met throughout the screening process to resolve conflicts and discuss any uncertainties related to study selection [20].

### 2.5. Data Charting

A form was developed by the authors to extract study characteristics. The characteristics of each full-text article were extracted by one independent reviewer (Steven Lam). The data collection categories included: author, year of publication, study location, study design, waste management practice, and key results. The data were compiled in a single spreadsheet using DistillerSR report function and subsequently imported into Microsoft Excel 2010 (Microsoft Corporation, Redmond, WA, USA).

**Table 2:** Inclusion and exclusion eligibility criteria applied during screening of articles to identify articles examining the human health risks of wastewater, human excreta, and animal excreta management practices in Southeast Asia.

Inclusion	Exclusion
-Described wastewater and excreta management practices in relation to human health, environmental health, or perceived health risks	-Described wastewater and excreta management practices without reporting poor health outcome, or described poor health outcomes with no association to wastewater or excreta management practices
-Study conducted in a country in Southeast Asia	-Study conducted outside of Southeast Asia
-Article published in the English language	-Article published not in the English language
-Original research in a peer-reviewed journal	-Workshop proceedings, reviews, letters to the editor, abstracts
-Studied poor health outcomes in human beings	-Reports on plants, animals, and *in vivo* or *in vitro* populations

### 2.6. Summarizing and Reporting

A narrative synthesis approach was used to provide an overview of the existing literature. Firstly, an overall summary of study findings was synthesized taking into account study variations that may affect generalizability of research results, such as variations in populations, wastewater and excreta management practices. Then, study results were organized into categories using thematic analysis techniques [21]. While scoping reviews generally do not assess quality of included studies, some argue that scoping reviews should include a quality assessment phase [20]. As such, in this paper, we considered and evaluated the study designs used in each article.

## 3. Results

### 3.1. Overview of Studies Identified

**Figure 1:**
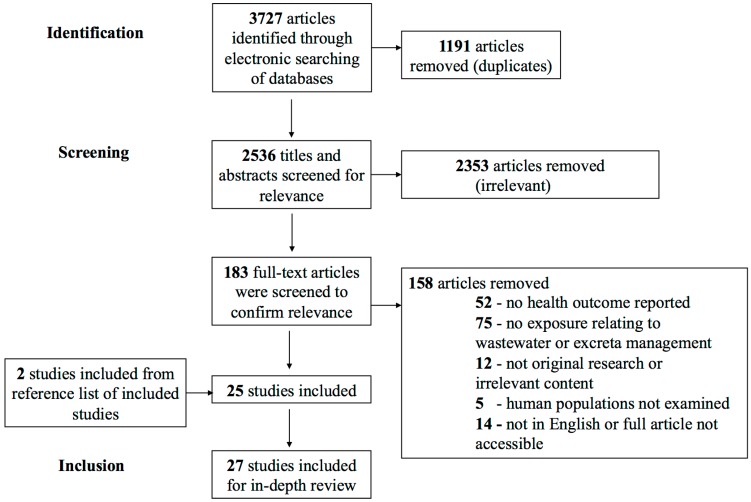
Flow chart of the selection of studies that examined the human health risks of wastewater, human excreta, and animal excreta management practices in Southeast Asia.

The search strategy identified 1126 studies in PubMed, 1319 studies in CAB Direct, and 1282 in Web of Science, totaling 3727 articles. Duplications were removed, resulting in 2536 unique citations. After primary title and abstract screening, 183 were included as potentially relevant. After examination of the full text of these articles, 25 articles met the inclusion criteria. The hand-search of reference lists from the included articles resulted in the addition of 2 more articles. The inter-rater reliability for title/abstract article screen and full-text article screen was 0.61 and 0.72, respectively, indicating good agreement [22]. Titles and abstracts were most often considered not relevant because the study was not conducted in the defined geographic area (Figure 1). The majority of studies were conducted in Vietnam (n = 24) with other studies conducted in Cambodia (n = 1), Laos (n = 1), and Thailand (n = 1). The articles reviewed represented a variety of study designs including cross-sectional (n = 11) [23,24,25,26,27,28,29,30,31,32,33], cohort (n = 4) [34,35,36,37], case-control (n = 3) [38,39,40], sampling and testing (n = 4) [41,42,43,44], risk assessment (n = 2) [45,46], and qualitative research (n = 3) [3,7,47].

The studies explored associations between wastewater and excreta management practices and human health (Table 3). Poor health outcomes through occupational exposures explored in the studies included diarrhea [34,35,38,45], skin infection [32,33,36,37], parasitic infection [23,24,25,26,27,28,29,30,39,44], bacterial infection [41,42,46], heavy metal exposure [43], and epilepsy [40]. Only a few studies explored perceived health risks including unpleasant odour and skin infection [3,7,31,47]. Other potential exposure routes included the consumption of contaminated fish, vegetables, and fruits [24,42,43,44,45,46].

**Table 3:** Description of relevant studies on human health risks of wastewater and excreta management practices to farmers, consumers and community members in Southeast Asia.

Author/Year	Country	Year/(Study)	Target Group	Practice	Study Design *	Health risk and Conclusions
Diarrhea Disease Studies						
Pham-Duc *et al.* 2014	Vietnam	2009	Adult farmers	Wastewater and excreta	Cohort Case-control	The incidence rate of diarrhea was 28 episodes per 100 person-years at risk. Significant risk factors of diarrhea include eating raw vegetables (odds ratio [OR] = 2.4, 95% confidence interval [CI] 1.2–4.6), direct contact with wastewater (OR = 2.4, 95% CI 1.2–4.7), and handling of human excreta (OR = 5.4, 95% CI 1.4–21.1) and animal excreta (OR = 3.3, 95% CI 1.8–6.0).
Trang *et al.* 2007	Vietnam	2002	Adults	Wastewater	Cohort Case-control	The incidence rate of diarrhea was 28.1 episodes per 100 person-years at risk. Wastewater contact was a significant risk factor for diarrhea (OR = 1.98, 95% CI 1.18–3.3).
Hien *et al.* 2007	Vietnam	2002	Children	Wastewater	Case-control	Wastewater contact was a risk factor for diarrhea.
Ferrer *et al.* 2012	Thailand	N/A	Farming households	Wastewater	Quantitative microbial risk assessment	Canal water and vegetables were heavily contaminated with *Giardia lamblia* and *Entamoeba histolytica*. Infection risk was high in tested scenarios and largely exceeded the acceptable risk given by World Health Organization guidelines.
Parasitic Infection Studies						
Pham-Duc *et al.* 2013	Vietnam	2008	Farming households	Wastewater and excreta	Cross-sectional	Contact with wastewater was a significant risk factor for helminth infection (OR = 1.5, 95% CI 1.1–2.2) and *Ascaris lumbricoides* infection (OR = 2.1, 95% CI 1.4–3.2). Significant risk factors for *Trichuris trichiura* infection include the use of human excreta (OR = 1.5, 95% CI 1.0–2.3).
Yajima 2009	Vietnam	2007	Community members	Human excreta only	Cross-sectional	Consumption of vegetables fertilized with human excreta → high helminth infection rate.
Trang *et al.* 2006	Vietnam	2003	Farming households	Wastewater	Cross-sectional	No significant association was found between wastewater exposure and helminth infections.
Trang *et al.* 2007	Vietnam	2002	Adults and children	Wastewater and human excreta	Cross-sectional	Wastewater exposure did not pose a significant risk for helminth infection. Significant risk factors for helminth infections include lack of sanitation facilities (relative risk [RR] = 1.97, 95% CI 0.95–4.09) and use of fresh or inadequately composted human excreta (RR = 1.19, 95% CI 0.93–1.53).
Nguyen *et al.* 2006	Vietnam	1995	Women	Excreta	Cross-sectional	76% of Vietnamese women were infected with helminth. The use of untreated feces as fertilizer was significantly associated with *A. lumbricoides* (OR = 1.2, 95% CI 1.0–1.6).
Verle *et al.* 2003	Vietnam	2003	Community members	Human excreta only	Cross-sectional	Eggs of parasitic species were detected in 88% of stool samples. While it was mentioned that human excreta was commonly used as fertilizer, the epidemiological linkage between waste management practice and parasitic infection was not explored.
Olsen *et al.* 2006	Vietnam	2004	Adult farmers	Excreta	Cross-sectional	81.8% prevalence of helminth infection. While it was mentioned that human excreta and wastewater were commonly used as fertilizer, the epidemiological linkage between waste management practice and parasitic infection was not explored.
Van der-hoek *et al.* 2003	Vietnam	1990	Community members	Human excreta only	Cross-sectional	44.4% prevalence of helminth infection. The use of human excreta as fertilizer was a significant risk factor for hookworm infection, especially among adult women.
Pham-Duc *et al.* 2011	Vietnam	2008	Community members	Wastewater and excreta	Case-control	Personal hygiene factors determined infection with *E. histolytica*, rather than exposure to human and animal excreta in agricultural activities.
Uga *et al.* 2009	Vietnam	N/A	Community members	Excreta	Sampling, microbial testing, surveys	Vegetables purchased at a market in Vietnam were highly contaminated with parasite eggs excreted by animals and humans.
Bacterial Infection Studies						
Yajima and Kurokura 2008	Vietnam	2007	Fish	Animal excreta only	Sampling, quantitative microbial risk assessment	Direct use of animal excreta was a major contributor to fecal contamination of pond water and skin of cultured fish. Estimated risks of enteric infection through farming activities and fish handling were 100–1000 times higher than the US Environmental Protection Agency acceptable risk.
Ha *et al.* 2008	Vietnam	2006	Farming households	Wastewater	Sampling and microbial testing	Risk of *Escherichia coli* infection from vegetables as vegetables were washed in nearby canals (contaminated with human and animal excreta) after harvesting.
Huong *et al.* 2014	Vietnam	2011	Farmers	Use of biogas effluent	Sampling and questionnaire	There was potential exposure of fruits and vegetables to *enterococci, E. coli, Clostridium perfringens, and Salmonella* through use of biogas effluent in agriculture.
Skin Infection Studies						
Trang *et al.* 2007	Vietnam	2004	Farmers	Wastewater	Cohort	Exposure to wastewater was a significant risk factor for skin diseases (RR = 1.89, 95% CI 1.39–2.57).
Trang *et al.* 2007	Vietnam	2002	Community members	Wastewater	Cohort Case-control	The incidence rate of skin ailments was 32.5 episodes per 100 person-years at risk. Exposure to wastewater was significantly associated with skin infections (OR = 2.74, 95% CI 1.29–5.82).
Anh *et al.* 2007	Vietnam	2005	Farmers	Wastewater	Cross-sectional	Contact with wastewater was a significant risk factor for dermatitis (OR = 3.0, 95% 1.1–7.7).
Anh *et al.* 2009	Cambodia	2004	Community members	Wastewater	Cross-sectional	Occupational exposure to wastewater was not significantly associated with dermatitis.
Other Health Risk Studies						
Tran *et al.* 2007	Laos	N/A	Patients with epilepsy	Human excreta only	Case-control	The use of human feces to fertilize domestic vegetable gardens was significantly associated with epilepsy (OR=4.9, 95% CI 1.1–22.1).
Marcussen *et al.* 2007	Vietnam	N/A	Fish (common carp, silver carp, tilapia)	Wastewater	Sampling	The consumption of common carp, silver carp and tilapia produced in wastewater-fed ponds of Hanoi seemed not to be a food safety problem with respect to arsenic, cadmium, and lead.
Perceived Health Risk Studies						
Pham-Duc *et al.* 2006	Vietnam	2003	Farming households	Human excreta only	Qualitative	Farmers were at risk to pathogens in excreta through improper handling practices.
Jensen *et al.* 2008	Vietnam	2004	Farmers	Human excreta only	Qualitative	The community did not associate risks with the use of composted excreta if it was dry and lacked odour.
Knudsen *et al.* 2008	Vietnam	N/A	Farmers	Wastewater and human excreta	Qualitative	Farmers perceived health risks of wastewater as non-serious (skin problems) and “smelly feces” as serious (polluted air).
Anh *et al.* 2007	Vietnam	2004	Farming households	Wastewater	Cross-sectional	Exposure to wastewater was a perceived as a risk factor for skin problems.

* The study design was determined by the original article’s reported study design.

**Figure 2:**
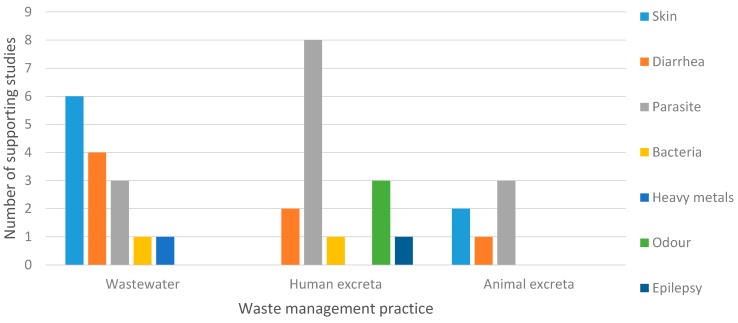
Health risks from wastewater, human excreta and animal excreta management practices and number of supporting studies.

Wastewater and human excreta management practices were more often investigated rather than animal excreta management practices (Figure 2). Health risks studies on wastewater included diarrhea [34,35,38,45], parasitic infection [23,25,26], skin infection [7,31,32,33,36,37], bacterial infection [42], and heavy metals exposure [43]. Health risk studies on human excreta include diarrhea [34,39], parasitic infection [23,24,26,27,28,29,30,44], unpleasant odour [3,7,47], bacterial infection [41], and epilepsy [40]. Health risk studies on animal excreta include diarrhea [34], parasitic infection [26,28,43] and bacterial infection [40,45].

Studies reported differences in poor health outcomes between males and females. Six studies reported no significant difference in health outcomes between males and females [23,24,28,31,33,36]. In seven studies, poor health outcomes were significantly higher for females than males [22,25,27,29,32,34,35]. In one study, poor health outcomes were significantly higher for males than females [30]. In three studies, the difference in health outcomes between males and females was not explored or statistical analysis was not reported [26,37,38].

The degree of risk of obtaining poor health outcomes varied between studies. Some studies suggested that the lack of sanitation facilities or personal hygiene factors were risk factors for poor health outcomes [24,25,38], while most studies suggested that direct exposure to wastewater or excreta in agricultural activities was a risk factor for poor health outcomes [22,26,29,30,31,33,35,36,39,45], or a combination of both [34]. Some studies reported the agricultural use of excreta was not correlated with any poor health outcomes [23,32].

**Figure 3:**
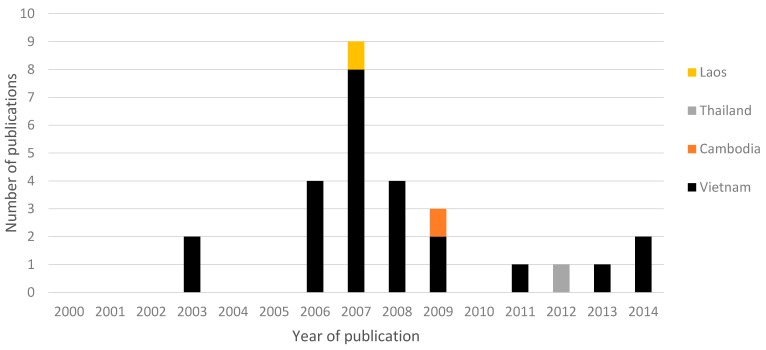
Number of publications on wastewater and excreta management practice on human health in Southeast Asia from year 2000 to 2014.

Of the studies included, 24 out of 27 (89%) were conducted in Vietnam (Table 3 and Figure 3). The majority of articles were published between 2006 and 2009, with a low number of publications published from 2011 onwards (Figure 3). From 2006 to 2009, eight out of 20 studies made references to the recently revised “Guidelines for the Safe Use of Wastewater, Excreta and Greywater” in agriculture and aquaculture published by World Health Organization (WHO) in 2006 [7,25,30,31,32,35,36,37]. Many studies argued that while risk for some diseases are well established (e.g., helminth infections), the information on other potential human health risks are largely anecdotal (e.g., skin infections, diarrhea) or limited, hence the need to conduct further studies on health risks [24,25,26,30,31,34,35,36,46].

### 3.2. Poor Human Health Outcomes and Risk Factors

#### 3.2.1. Diarrheal Disease Including Parasitic Infections

Four studies including case-control and cohort studies explored associations between wastewater and excreta management practices and diarrheal disease [33,34,37,44]. Through multivariable analysis, significant risk factors associated with diarrhea in adults included consumption of raw vegetables and exposure to wastewater and excreta [33,34]. In an area where the use of untreated wastewater in agriculture is common practice, a large variety of diarrheagenic *Escherichia coli* subtypes were found in children [37]. Through a quantitative microbial risk assessment, Ferrer *et al.* 2012 assessed diarrhea infection risks caused by the use of and contact with wastewater in Bangkok, Thailand [44]. The study found that canal water and vegetables were heavily contaminated with *Giardia lamblia* and *Entamoeba histolytica*, and infection risk was high in tested scenarios and largely exceeded the acceptable risk given by World Health Organization (WHO) guidelines [44].

Ten studies explored the association between wastewater and excreta management practices and parasitic infections [22,23,24,25,26,27,28,29,38,43]. Findings from four cross-sectional studies showed significant associations between helminth infections and use of wastewater and excreta in agriculture through multivariable analysis [22,25,26,29]. Of the participants in one study, 47% were infected with any helminth; people having close contact with wastewater had a higher risk of helminth infection and *Ascaris lumbricoides* infection compared to those without contact [22]. Of the participants in a different study, 39% were infected with any helminth [25]. In this study, wastewater exposure was not associated with an increased risk for helminth infection in this community; instead, the lack of sanitation facilities and use of fresh or inadequately composted human excreta in agriculture were important risk factors [25]. In a nationwide cross-sectional survey among women in Vietnam, 76% were infected with one or more helminth species [26]. Hookworm (*Necator americanus*) infection was associated with farming, and *A. lumbricoides* infection was associated with use of untreated feces as fertilizer [26]. Van der Hoek *et al.* explored the current status of soil-transmitted helminths in Vietnam; the prevalence of *A. lumbricoides* infection, *Trichuris trichiura* infection, and hookworm was 44.4%, 23.1%, and 28.6%, respectively [29]. Vegetable cultivation in which human excreta was used as fertilizer was a risk factor for hookworm infection, especially among adult women [29]. Vegetables purchased at a suburban market in Hanoi, Vietnam were examined for helminth eggs [43]. The study revealed that vegetables were highly contaminated with parasitic eggs and 81% of adult villagers interviewed stated that they used animal feces and human feces as fertilizer [43].

Findings from two cross-sectional studies did not find a significant association between helminth infection and wastewater use [23,24]. In one study, while the overall prevalence of infection with any helminth was 53.4%, the agricultural use of wastewater was not correlated with any infections [24]. Through multivariate analysis, poor sanitation and hygiene practices along with not using protective measures were important independent risk factors for helminth infection [24]. In the other cross-sectional study, at least one of the parasites was detected in 72.3% of the samples [23]. Through univariate analysis, the agricultural use of human feces (an occupational exposure) was not correlated with any of the infections. Rather, the authors suggested that the consumption of vegetables commonly fertilized with human feces (an oral-route exposure) had led to the high infection rates with *A. lumbricoides* and *T. trichiura* in the community [23].

Two cross-sectional studies explored the prevalence of soil-transmitted helminths [27,28]. In one study, about 82% were infected with at least one of the three main soil-transmitted helminths (*A. lumbricoides*, *T. trichiura*, hookworm) [28]. In the other study, eggs or cysts of at least one parasite species were detected in 88% of stool samples (n = 2,522) [27]. While these two studies mentioned the common use of human and animal feces as fertilizer in the study areas, the epidemiological linkage between waste management practice and health outcome was not explored. A case-control study in Ha Nam, Vietnam was conducted to assess risk factors for *E. histolytica* infection, where wastewater and excreta were commonly used in agriculture [38]. None of the factors related to the use of excreta were significantly associated with *E. histolytica* infection in this article. Where human and animal excreta and Nhue River water were intensively used in agriculture, socio-economic and personal hygiene factors were significantly associated with *E. histolytica* infection, rather than exposure to human and animal excreta in agricultural activities [38].

#### 3.2.2. Skin Infections

Four studies explored associations between skin disease and wastewater exposure through multivariable analysis [31,35,36]. Of the 1,103 participants in one study, 381 (35%) reported a skin problem, primarily dermatitis, and superficial fungal infections [36]. Exposure to wastewater was identified as a significant risk factor for skin disease [36]. In a cohort of 636 adults in Hanoi, Vietnam, the incidence rate of skin ailments was 32.5 episodes per 100 person-years at risk [35]. Independent determinants of skin ailments included wastewater contact in the past seven days, female gender, fish farming-related jobs, and lack of protective measures [35]. In a cross-sectional study with two follow-ups in Hanoi, Vietnam, the overall prevalence of dermatitis from 592 interviews was 6.3% [31]. The commune which used wastewater had a much higher overall prevalence of dermatitis (10.4%) than the commune that did not (2.1%; *p*-value < 0.001) [31]. This study showed that occupational wastewater contact was a significant risk factor for dermatitis [31]. A cross-sectional study by Anh *et al.* 2009 did not find a significant association between dermatitis and occupational wastewater exposure [32]. While the overall dermatitis prevalence was 6.1% among 650 household members, very few aquaculture workers applied personal protection and the factor had no significant effect on dermatitis [32].

#### 3.2.3. Bacterial Infections

Three studies explored bacterial infections linked to wastewater or excreta management practices [40,41,45]. Ha *et al.* explored potential routes of bacterial infection via consumption of raw vegetables through sampling and microbial testing [41]. Vegetables in the markets and restaurants had higher total coliforms and *E. coli* counts than the vegetables at the vegetable cultivation fields [41]. In search of the potential contamination sources, it was found that vegetables were washed in nearby wastewater canals after harvesting [41]. Huong *et al.* evaluated the survival of *Salmonella spp.* and fecal indicator bacteria in biogas digesters through sampling and questionnaires about waste management practices in 12 farms in Ha Nam, Vietnam [40]. Results showed that the concentration of enterococci, *E. coli*, and *Clostridium perfringens* spores was overall reduced by only 1–2 log_10_-units in the biogas digesters compared with raw slurry and biogas effluent, and *Salmonella spp.* was found in both raw slurry and biogas effluent. *Salmonella spp.* also showed resistance to several antimicrobials tested. The study concluded that there was potential exposure to fecal bacteria and *Salmonella* through the use of biogas water for irrigation of produce [40]. A study investigated the use of animal manure and fecal contamination of pond water and fish skin, as well as investigated the risk of enteric infection using quantitative microbial risk assessment [45]. *E. coli* counts on fish skin were higher than acceptable standards, and the direct use of wastewater and animal manure were risk factors for enteric infection through fish farming and handling activities [45].

#### 3.2.4. Other Relevant Health Studies

There were a few studies that investigated epilepsy and heavy metal exposure from wastewater and excreta management activities. One study assessed the major etiologic categories of epilepsy in a rural district of the Lao People’s Democratic Republic through a case-control study. Matched analysis determined that the use of human feces to fertilize domestic vegetable gardens were significantly associated with epilepsy (OR = 4.9, *p* = 0.04) [39]. Marcussen *et al.* investigated the food safety of fish from production systems in Hanoi. The consumption of common carp, silver carp and tilapia produced in wastewater-fed ponds of Hanoi seemed not to be a food safety problem with respect to arsenic, cadmium, and lead [42].

#### 3.2.5. Perceived Health Risks

Two studies explored health risk perceptions of human excreta management practices [3,46]. In one study in Central Vietnam, farmers perceived human excreta to be very dirty and containing pathogens; however, once composted and the unpleasant odour was reduced, farmers perceived that excreta could be safely handled [46]. In another study in Central Vietnam, farmers emphasized that human excreta could be harmful to health and mentioned that contact with human excreta could cause diarrhea and intestinal diseases, as well as lung diseases [3]. It was highlighted that the bad smell (‘*mùi hôi*’ in Vietnamese) coming from latrine waste presented a health risk when handling excreta, and the community did not associate risks with the use of composted excreta if it was dry and lacked odour [3].

Anh *et al.* explored perceived health problems of wastewater through a cross-sectional study of 3089 households in Phnom Penh, Hanoi, and Ho Chi Minh City. The most important health problem relating to wastewater exposure was skin problems, reported by 4% of all people surveyed. The study suggested that the exposure to wastewater can be a risk factor for skin problems [30]. Knudsen *et al.* explored Vietnamese farmers’ health risk awareness, knowledge, and practices related to their use of wastewater and human excreta [7]. Farmers perceived health risks of wastewater as non-serious (e.g., skin problems) and “smelly feces” as serious (e.g., polluted air). Farmers mainly considered hygiene and health as a women’s issue, thus men’s responsibility for the health and hygiene of the family should be emphasized in health promotion activities [7].

## 4. Discussion

Our review summarized and synthesized the results from 27 studies investigating the human health risks associated with wastewater and excreta management practices in Southeast Asia. The three types of waste used in agriculture presented subtle but important differences in health outcomes (Figure 2), which poses challenges in summarizing data and generalizing results. Overall, the human health risks associated with wastewater and excreta management practices included skin infection, parasitic infection (predominately helminths including hookworm, *A. lumbricoides, T. trichiura*), bacterial infection, and diarrhea, which are consistent with other studies from other regions on wastewater and excreta reuse without proper waste treatment [47,48,49]. The WHO estimates that 2.2 million people die annually from diarrheal diseases and that 10% of the population in the developing world are infected with intestinal worms related to improper wastewater and excreta management [47]. Other contaminants in wastewater and excreta of public health concern, such as excess nutrients, antibiotic residues, growth promoters, hormones, hazardous chemicals, or heavy metals [8,12,17], were not well captured in our review, presenting opportunities for future research. One study found an associated between epilepsy and using human feces as fertilizer in the vegetable garden [39]. A risk factor for cysticercosis infection (serology of epilepsy) is exposure to human feces with *Taenia* eggs through food products [50]. More studies are needed to confirm epilepsy as a potential health risk to wastewater and excreta management practices.

Nearly half of the study designs were cross-sectional, in which exposure and outcome were assessed at the same point in time. This study design posed limitations in drawing causal inference from the results as these studies could not determine whether exposure to wastewater or excreta occurred before, during, or after the onset of the poor health outcome. The cohort studies had large sample sizes ranging from 636 to 1,103 individuals, had a follow-up duration of 12 to 16 months, and explored either skin disease [35,36] or diarrhea associated with wastewater and excreta management practices [33,34]. The case-control studies ranged from 46 to 232 cases [33,37,38,39] and explored *E. histolytica*, diarrhea, epilepsy, or helminth infections associated with wastewater and excreta management practices. While there appears to be associations between wastewater and excreta management practices (e.g., insufficient treatment practices, lack of safety equipment used) and health effects (e.g., skin disease, diarrhea), there is a need for more longitudinal study designs, to support findings of our study.

Some studies showed no difference in poor health outcomes between males and females while others showed differences, with women being more susceptible to infections. This divergent finding may be due to gender role differences in wastewater and excreta management in different communities, including differences in practices or exposure risks (e.g., frequency, intensity of exposure). The risks to women were not given much consideration in the literature from Southeast Asia. Given the trend towards more women engaging in agriculture than men [51], understanding the practices and perceptions of wastewater and excreta management among women are important. Considering men’s perspective on wastewater and excreta management is also important to address in health promotion activities considering one study found that male farmers typically considered hygiene and health as women’s issues, in the sense that women represented the family’s health and therefore it was more important that women protect their own health such as using protective equipment when handling waste [7]. Future research is recommended to determine if there is an association between female gender roles and increased risk for poor health outcomes, perhaps through a meta-analysis, a useful quantitative method that allows integrating results from independent studies with similar characters and to test the analyzed data for statistical significance.

The selected studies mainly focused on poor health outcomes associated with wastewater or human excreta, but not animal excreta. The increasing volumes of animal excreta and contaminants present have a very high potential of causing environmental and public health hazards, if not properly managed [8,52]. Excreta management practices, such as treatment through biogas systems, may not adequately reduce microbial pathogens to acceptable levels [40]. Given the common practice of using animal excreta as fertilizer in agriculture, more epidemiological studies identifying poor health outcomes associated with animal excreta management practices and tracing the fate of pathogens in animal waste treatment systems and their surrounding environments are recommended [8,12].

It was emphasized that the consumption of contaminated fish, vegetables, and fruits were potential exposure routes for poor health outcomes. While studies suggested that vegetables and fruits were contaminated through wastewater or excreta management practices, the link to human health outcomes was not explored. Until recently, environmental health studies also explored wastewater and excreta treatment practices and their capacities to reduce bacterial load [40], with discussion surrounding health implications but without exploring linkages. Recognizing some of these study limitations, recommendations for future studies include: studies determining the source of contamination of fruits and vegetables; and epidemiological studies conducted in conjunction with microbial analysis studies to explore the poor human health outcomes associated with wastewater and excreta management practices or waste-contaminated products.

The health risks associated with wastewater and excreta management practices were inconsistent among studies and, although present, may be low. For instance, a study in Laos did not report associations between dermatitis and wastewater exposure [32], unlike similar studies in Vietnam where wastewater exposure was a risk factor for dermatitis [7,31,35,36]. Despite inconsistencies among findings between studies, personal protection methods seem to be important for prevention of skin disease [32]. While most studies reported direct exposure to wastewater or excreta in agricultural activities was a risk factor for poor health outcomes, some studies suggest that direct exposure was not a risk factor, but personal hygiene factors are [24,38]. Indeed, in a couple studies, the lack of safe handling practices exacerbated the risk of poor health outcomes [33,34].

Only a few studies analyzed local perceptions of health risks associated with wastewater and excreta management practices [3,7,30,46]. The perceived health risks included unpleasant odour and skin infections, consistent with other health perception studies in other countries [53,54]. Without a clear understanding of perceptions of health risks associated with wastewater and excreta management systems, it is difficult to assess risks because risks perceptions and related hygiene behavior are an essential component of the health risk assessment [7]. There was also limited studies on experts or community leader’s perceptions of risk [7,46]. It is generally believed that experts think about wastewater and excreta management differently than members of the general public [55]. Experts provide advice to farmers on how to manage waste as well as contribute to decision-making surrounding waste management. The implications of differing perceptions on wastewater and excreta management practices have not yet been explored. We recommend that studies are conducted to understand risk perceptions of farmers, community members, researchers and decision-makers.

In many cases, the assessment of impacts of wastewater and excreta management on health and environment was often considered in isolation. For instance, some studies assessed the impact of waste management practices on human health but did not consider impact on the environment [33,34,37]. Others explored the link between wastewater and excreta management practices on the environment but did not consider impact on human health [23,40]. It is important that human and environmental health are considered together, as one study reported that the agricultural use of waste was not correlated with poor health outcomes, but rather the consumption of vegetables commonly fertilized by waste was [23]. A more integrated or systems approach to waste assessment which addresses the interactions of human, animals and environment are recommended [56,57].

Most of the studies (89%) qualifying for inclusion in our report focused on study sites in Vietnam, despite the common practice of using wastewater, animal and human excreta in many other countries in Southeast Asia. Only a few articles on wastewater and excreta management were published from other countries [32,39,44]. As such, the conclusions of this study may not be applicable to other countries in Southeast Asia.

Many studies in this review referred to the “2006 WHO Guidelines for Safe Use of Wastewater, Excreta, and Greywater in Agriculture and Aquaculture” and in 2015, the WHO created the “Sanitation Safety Planning,” a step-by-step risk based approach to assist in the implementation of the 2006 WHO guidelines [58]. A book was also created to guide through the WHO guidelines and assess for health risks of wastewater and excreta management [59]. Until recently, regulatory measures were often incompatible with local settings where most wastewater and excreta use takes place due to rigid guideline values [60]. Incorporating insights from this study, in particular the health risks of wastewater and excreta management in Southeast Asia, can provide a Southeast Asia context and improve uptake of WHO guidelines.

Agricultural related risk factors including exposure to excreta for fertilizing fields or exposure to wastewater for irrigation are among the important determinants of health risks. Public health interventions supporting adequate sanitation and proper hygienic handling and use are required to address these risk factors. While alternatives to reusing excreta or wastewater for agricultural purposes exist (e.g., chemical fertilizers), reusing excreta and wastewater saves expenditure for chemical fertilizers, improves soil fertility, and saves natural resources by recycling nutrients. Future research may consider health risks of using chemical fertilizers compared to excreta and testing innovative solutions to mitigating health and environmental risks.

### Limitations

We noted several benefits from adopting a scoping review methodology. We found the scoping review to be a useful approach for identifying evidence for public health risks of wastewater and excreta management in a transparent way. The review also identified several knowledge gaps. Considering the many different languages used in Southeast Asia (e.g., Vietnamese, Lao, Thai, Burmese, Khmer, Malay, Indonesian) and to limit our study scope, the study was restricted to only English language, peer-reviewed publications. As such, there may be more relevant articles and reports, in other languages, or in ‘grey literature’, that were missed in this study. For instance, a hand-search of two popular Vietnamese public health journals resulted in five relevant papers, which were not summarized in our report (See Supplementary Material). Future systematic literature reviews on this topic should consider the inclusion of articles in other languages and also the protective health effects of wastewater and excreta management. Further, to limit the number of articles returned in our search to a feasible number, we did not consider environmental health search terms in the search strategy (e.g., air quality, water quality, soil quality) since human health was our main outcome of interest. The impact of waste management on environmental health should be considered more comprehensively in future systematic literature reviews.

Scoping reviews, by definition, were not intended to assess the quality of the literature scoped; therefore, it is difficult to summarize actual evidence on health risks. For some studies, we noted observations on study designs, especially regarding gender analysis and linking environment and human health components, so that future research can address these observations and strengthen evidence of health risks. While the published material captured in our review does not necessarily provide sufficient evidence to base decisions, it provided insight on potential health risks and identified research gaps.

## 5. Conclusions

We have conducted a scoping review of the literature to explore evidence of health risks of wastewater and excreta management practices in Southeast Asia. While there were some insights drawn surrounding potential health risks of wastewater and excreta management (e.g., skin infection, diarrhea, parasitic infection, bacterial infection), concerns remain about the actual health risks due to lack of rigor study designs. Longitudinal studies would provide more persuasive evidence of health risks of wastewater and excreta management than cross-sectional studies. Further, some studies suffered from limitations related to lack of exposure assessment and inclusion of gender perspectives. Epidemiological studies on health risks of wastewater and excreta management that incorporate characterization of exposures and consideration of gender roles are recommended.

Agricultural related risk factors including exposure to excreta for fertilizing fields or exposure to wastewater for irrigation are among the important determinants of health risks. Public health interventions supporting sanitation and proper hygienic handling and use are needed to address these risk factors. While alternatives to reusing excreta or wastewater for agricultural purposes exist (e.g., chemical fertilizers), reusing excreta and wastewater saves expenditure for chemical fertilizers, improves soil fertility, and saves natural resources by recycling nutrients. Future research may consider health risks of using chemical fertilizers compared to excreta and testing innovative solutions to mitigating health and environmental risks.

## Supplementary Files

Supplementary File 1
