# Supplementary material for: Evidence for Public Health Risks of Wastewater and Excreta Management Practices in Southeast Asia: A Scoping Review
Source: Int J Environ Res Public Health. 2015 Oct 15;12(10):12863–85. doi: 10.3390/ijerph121012863 (PMC4627004; doi:10.3390/ijerph121012863)
Supplement: Supplementary File 1 [file ijerph-12-12863-s001.pdf]

Evidence for Public Health Risks of Wastewater and Excreta Management Practices in Southeast Asia: A Scoping Review

Table S1. Relevant Vietnamese articles hand-searched from Vietnam Journal of Public Health and Vietnam Journal of Preventative Medicine.

Author/ Year	Area of study	Year (study)	Target Group	Practice	Study Design	Health risk Concluded from Study
Khuong *et al.* 2011	Kim Bang, Ha Nam Province	2009	People engaged in wastewater or excreta management	Wastewater and excreta	Quantitative microbial risk assessment	Occupational exposure to wastewater and excreta resulted in diarrhea risk by *Escherichia coli* to be 11 times, by *G. lamblia* to be 210 times, and by *C. parvum* to be 3 times higher than the acceptable risks proposed by WHO.
Dam *et al.* 2011	Ha Nam Province	2009	People engaged in wastewater management	Wastewater	Microbial testing	*E. coli* counts in samples of wastewater exceeded standard values for agricultural use; Humans may be at risk to E. coli infection through wastewater
Tu *et al.* 2011	Hoang Tay and Nhat Tan commune, Kim Bang, Ha Nam Province	2008	Community members	Wastewater	Cross-sectional	Farmers perceived health risks of wastewater as non-serious (e.g., skin problems) so they didn't use protective equipment; consuming fishes and vegetable from Nhue river may cause diarrhea.
Thanh *et al.* 2011	Hoang Tay and Nhat Tan commune, Kim Bang, Ha Nam Province	2005	Head of household	Wastewater	Cross-sectional	The results showed that using dirty water and unhygienic latrines would increase the risk getting disease (e.g., diarrhea, helminth, skin, gynecological, food poisoning, and sore eyes).

Table S1. *Cont*.

Author/ Year	Area of study	Year (study)	Target Group	Practice	Study Design	Health risk Concluded from Study
Trang *et al.* 2011	Hanoi and Nam Dinh	N/A	People engaged in wastewater management	Wastewater	Case-control	Wastewater exposure, poor sanitation and personal hygiene were risk factors for diarrhea disease. Risk factors for skin diseases include wastewater contact, wastewater-related cultivations, being female and absence of personal protective equipment.

References

1. Trang, D.T.; Hien, B.T.T.H.; Cam, P.D.; Tuan, N.D.; Molbak, K.; van der Hoek, W.; Dalsgaard A. 2011. Health risks associated with wastewater use in agriculture and aquaculture in periurban areas in Vietnam. *Vietnam J. Public Health* **2011**, *22*, 21–28.
2. Khuong, N.C.; Bich, T.H.; Phuc, P.D.; Viet-Hung, N. Assessment of diarrhea risk by microorganisms in wastewater and excreta used in agriculture in Hanam. *Vietnam J. Public Health* **2011**, *22*, 14–20.
3. Tranh, N.H.; Minh, H.V.; Hung-Viet, N. Relationship between self-reported health status and the condition of water and sanitation in Hoang Tay and Nhat Tan communes, Kim Bang district, Ha Nam province. *Vietnam J. Public Health* **2011**, *22*, 46–52.
4. Dam, L.; Hung-Viet, N.; Cam, P.D.; Mollby, R. The spread and antibiotic resistance of Escherichia coli from wastewater in Hanam. *Vietnam J. Public Health* **2011**, *22*, 37–45.
5. Tu, V.V.; Huong, N.T.; Phuc, P.D.; Hung-Viet, N.; Zurbrugg C. Developing a questionnaire to measure awareness and behaviours of people in relation to wastewater use in agriculture at Hoang Tay commune and Nhat Tan commune, Ha Nam province following Protection Motivation Theory. *Vietnam J. Public Health* **2011**, *22*, 67–72.

© 2015 by the authors; licensee MDPI, Basel, Switzerland. This article is an open access article distributed under the terms and conditions of the Creative Commons Attribution license (http://creativecommons.org/licenses/by/4.0/).
